# Electromagnetic Interference in Implantable Rhythm Devices: Comment

**Published:** 2002-10-01

**Authors:** PV Ramachandran

**Affiliations:** Professor of Radiology, Medical College Calicut, Kerala, India

This is a short comment on the editorial on Electromagnetic Interference in Implantable Rhythm Devices - The Indian Scenario in the July-September issue of the journal [1]. Regarding the statement: "Systems working at 0.5 Tesla are available in the country so that it may be considered in such situations…" , though the author conveys his point, it gives reader a feeling that 0.5 Tesla magnetic resonance imaging (MRI) units are advantageous. In fact for every other diagnostic purpose we would like to have higher Tesla MRI units. The 0.5 Tesla unit is inferior compared to higher Tesla systems. The cry of Eastern European countries in a conference held in November 2001 at Antwerpen by the European Working Group on Management in Radiology (EWGMR), was that they have only 0.5 Tesla magnets and so they can not do this , that , no functional MRI , no stroke imaging etc etc… But a western European reader can get confused. I do not think there will be a single system working at 0.5 Tesla in countries like Germany.
